# Serum fibroblast growth factor 19 and endogenous islet beta cell function in type 2 diabetic patients

**DOI:** 10.1186/s13098-019-0475-1

**Published:** 2019-09-24

**Authors:** Meng-jie Tang, Jian‑bin Su, Tian-li Xu, Xue‑qin Wang, Dong-mei Zhang, Xiao-hua Wang

**Affiliations:** 1 Department of Endocrinology, Affiliated Hospital 2 of Nantong University and First People’s Hospital of Nantong City, No. 6 North Hai-er-xiang Road, Nantong, 226001 China; 2 Department of Clinical Laboratory, Clinical Medicine Research Center, Affiliated Hospital 2 of Nantong University and First People’s Hospital of Nantong City, No. 6 North Hai-er-xiang Road, Nantong, 226001 China; 3 Medical College of Nantong University, No. 19 Qi-xiu Road, Nantong, 226001 China

**Keywords:** Islet beta cell function, Insulin sensitivity, FGF19, Type 2 diabetes

## Abstract

**Background:**

Fibroblast growth factor 19 (FGF19) takes part in maintaining the balance of glycolipids and may be involved in regulating the secretory activity of islet beta cells in patients with type 2 diabetes. This study aimed to evaluate the relationship between the levels of serum FGF19 and endogenous islet beta cell function in type 2 diabetic patients.

**Methods:**

Samples were obtained from 271 subjects: 85 drug-naïve type 2 diabetes participants exclusively on lifestyle intervention (N-DM group), 122 type 2 diabetes subjects previously used medications (DM group) and 64 normal controls (NC group). Serum FGF19 concentrations were measured by ELISA. The insulin sensitivity (MI), insulin secretion (AUC_ins_/AUC_glu_) and insulin secretion-sensitivity index-2 (ISSI-2) were also measured in the N-DM and DM.

**Results:**

Serum FGF19 levels decreased, in order, from the NC group [median (interquartile range), 245.03 (126.23–317.43) pg/mL] to the N-DM group [170.05 (89.01–244.70) pg/mL] and, finally, to the DM group [142.25 (55.55–187.58) pg/mL] (*p* for trend < 0.05). Among subjects in the DM group, there was a positive trend in the serum FGF19 concentration; plasma insulin levels at 60 min, 120 min (INS60, INS120, respectively); and area under the insulin curve (AUC_ins_) at two points (*r *= 0.214, *p* = 0.025; *r* = 0.189, *p* = 0.048; *r* = 0.188, *p* = 0.049). However, the differences were no longer observed among the N-DM subjects. Simultaneously, the ISSI-2 was closely related to the serum FGF19 levels (*r* = 0.297, *p* = 0.002) among DM subjects. Furthermore, after adjusting for age, sex, duration, therapy and other clinical factors via multiple logistic regression analysis, ISSI-2 was a key independent factor in the levels of FGF19 (*β *=* 0.281*, *t *=* 2.557*, *p *=* 0.013*).

**Conclusions:**

The serum FGF19 level has a close relation with endogenous beta cell function among DM subjects, as assessed by the ISSI-2. As ISSI-2 is higher in N-DM group, FGF19 may be a main protector in dysfunction of beta cell.

## Background

Type 2 diabetes (T2D) affects up to 10% of the global adult population, causing a decline in human health. Insulin resistance and an islet beta cell defect are the main pathogenic mechanisms of diabetes and have severe complications, including microangiopathy and cardiovascular disease.

Fibroblast growth factor 19 (FGF19), a hormone expressed by ileal enterocytes, is a member of the FGF family involved in regulating lipid and nutrient metabolism [1–5]. The main physiological function of FGF19 is bile acid (BA) metabolism [6]. FGF19 (the mouse orthologue of which is called FGF15) binds to FGF receptor 4 (FGFR4) and generates physiological effects via the FGFR4-β-Klotho complex [7].

FGF19 has been widely discussed and has potential as a therapeutic target for metabolic disorders, including diabetes. In rodent model studies, FGF19 is able to decrease weight and improve metabolic disorders and insulin resistance [8–11]. In addition, FGF19 restores metabolic function in hormonal remodelling following bariatric surgery [12–14]. Clinical studies have observed that the insulin-independent beneficial effects of FGF19 in glucose metabolism can be explained by glucose effectiveness (GE) [15]. However, the aetiology of endogenous islet beta cell function is not fully understood, and not only insulin-independent but also insulin-dependent mechanisms may contribute to islet beta cell dysfunction. Glucose homeostasis is regulated through the BA-G-protein-coupled receptor 5 (TGR5)/FXR axis via incretin hormone: glucagon-like peptide 1 (GLP-1), which eventually stimulates the expression of FXR [16]. GLP-1 can regulate insulin secretion and glucose homeostasis by mimicking insulin action [17, 18]. However, the regulatory role of FGF19 in endogenous beta cell function among type 2 diabetic patients of different therapies remains insufficiently understood.

Our previous studies have shown that insulin sensitivity and insulin secretion were measured by the indices from oral glucose tolerance test (OGTT) results: insulin sensitivity, assessed by Matsuda Index (MI), insulin secretion, assessed by area under the insulin curve/area under the glucose curve (AUC_ins/glu_) [19]. Furthermore, the insulin secretion-sensitivity index-2 (ISSI-2), which evaluates integrated islet beta cell function, can be determined by the MI multiplied by the AUC_ins/glu_ [19–21].

Therefore, the current study was designed to identify the correlation between serum FGF19 and endogenous beta cell function in type 2 diabetic subjects with treatment before, naïve type 2 diabetic patients and normal controls. We also measured and compared the concentrations of FGF19 among these three groups.

## Methods

### Study design and subjects

The study was conducted during visits and follow-ups with patients from the Endocrinology Department at Affiliated Hospital 2 of Nantong University and First People’s Hospital of Nantong City from Apr 2017 to Oct 2018. The non-T2DM normal controls (NC) were required to be 20–60 years old and to have fasting plasma glucose < 6.1 mmol/L and 2-h plasma glucose (2hPG) < 7.8 mmol/L [1]. The inclusion criteria for newly diagnosed diabetic patients (N-DM) were being between 20 and 75 years old, having newly diagnosed type 2 diabetes as previously described [1] and being drug naïve exclusively on lifestyle intervention. The inclusion criteria for previously diagnosed diabetes patients (DM) were age between 20 and 75 years old, diagnosis of type 2 diabetes and with medications used. The exclusion criteria for all groups were type 1 diabetes, hyperthyroidism or hypothyroidism, severe hepatic disease, chronic renal insufficiency, cancer, acute diabetic complications, and current treatment with systemic corticosteroids. Finally, we recruited 64 NC, 122 DM and 85 N-DM participants. All participants gave written informed consent. The study was approved by the institution review board of Second People’s Hospital of Nantong and complied with the Declaration of Helsinki.

### Basic data collection

Upon enrolment, all participants were questioned and examined by experienced investigators to complete a questionnaire including parameters on their age, sex, weight, height, waist circumference, blood pressure, illness and medical therapy history. Body mass index (BMI) was calculated as the weight/the height squared. Blood pressure (BP) was tested in triplicate after at least 30 min of rest, and the average of three recordings was used for the analysis.

### OGTT procedures and calculation

After an overnight fast, all patients received OGTT (75 g of glucose) and plasma glucose at fasting (0) and 30, 60, 120 and 180 min (FPG, PG30, PG60, PG120, PG180, respectively) were determined. Measurements of serum insulin and C-peptide were also performed. The insulin sensitivity index was determined with the following equations: MI = 10,000/(FPG × fasting insulin × mean glucose × mean insulin)^1/2^ [2]. AUC_ins/glu_ was measured by area under the insulin curve/area under the glucose curve [2]. The ISSI-2 was calculated as the product of MI * AUC_ins/glu_ [2–4]. Insulin resistance was evaluated by HOMA-IR = FPG [mmol/L] × fasting insulin [mU/L]/22.5.

### Laboratory examination

Fasting blood samples were also collected to measure laboratory parameters. Measurements of serum insulin level, plasma glucose level, HbA1c concentration, low-density lipoprotein cholesterol (LDL-c), high-density lipoprotein cholesterol (HDL-c), total cholesterol (TC), and triglycerides (TG) were determined by standard laboratory procedures [5, 6]. Circulating FGF19 levels were detected by sandwich ELISA (FGF19 Quantikine^®^ DF1900; R&D Systems, Minneapolis, MN, USA). The range of the standard curve was 15.6–1000 pg/mL.

### Statistical analysis

Statistical analyses were conducted using SPSS software version 25.0 (Inc., Chicago, IL). Continuous variables with normal distribution are presented as the mean ± SD, skewed data as medians with interquartile ranges, and categorical variables as frequencies. All variables with skewed distributions were log transformed for further analysis. The skewness and kurtosis tests were used to assess the normality of the distribution. ANOVA/general linear model were conducted to compare inter-group differences between continuous variables, and the Pearson Chi-squared test was conducted to compare categorical variables. Moreover, a bivariate correlation analysis was used to select risk factors associated with FGF19. Then, multivariable linear regression analysis was undertaken to explore and identify independent contributors to FGF19. A *p* value < 0.05 was considered significant.

## Results

### Clinical characteristics of study participants

**Fig. 1:**
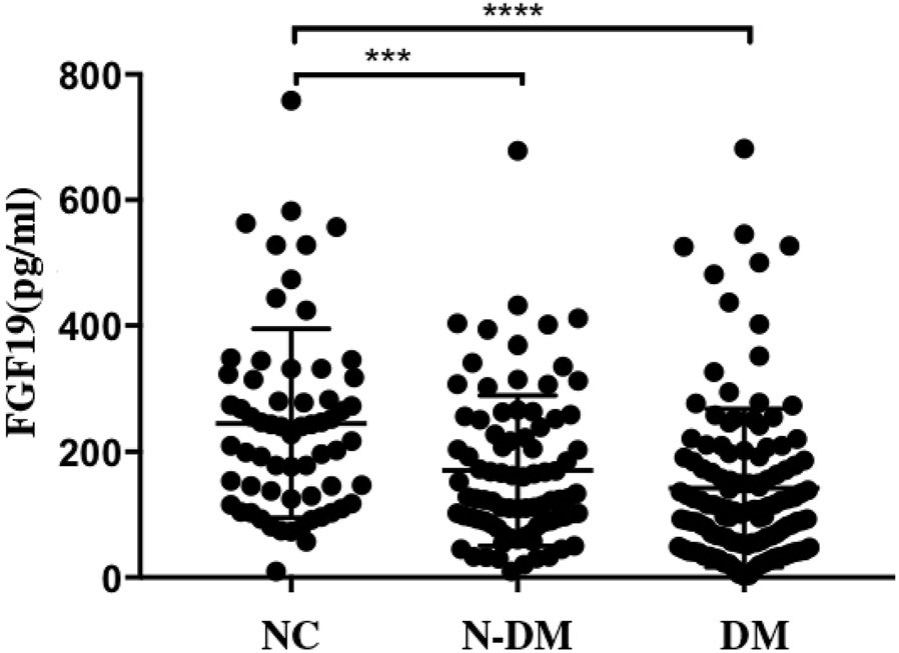
Comparison of serum FGF19 level across the three groups

**Fig. 2:**
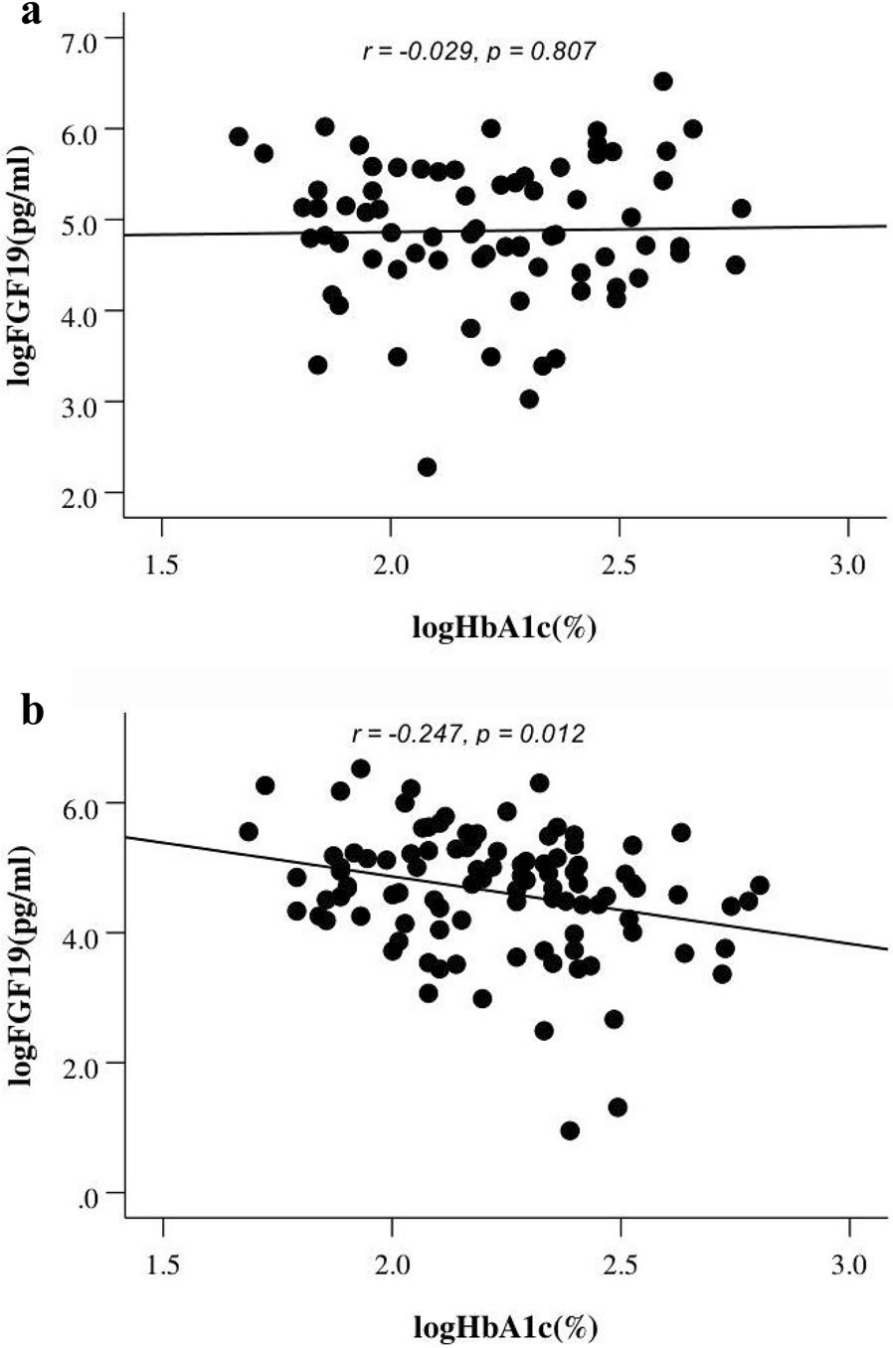
The relationship between HbA1c and fasting FGF19 among N-DM (**a**) and DM (**b**)

A total of 271 participants (mean age of 47.98 ± 13.188, 50.2% male) were recruited, and 195 subjects underwent an OGTT. We divided the population into NC group (n = 64), N-DM group (n = 85), and DM group (n = 122). Table 1 shows the clinical paraments of the three groups. Fasting FGF19 levels decreased in order from the NC group [245.03 (126.23–317.43) pg/mL] to the N-DM group [170.05 (89.01–244.70) pg/mL] and finally to the DM group [142.25 (55.55–187.58) pg/mL] (*p *<* 0.05*; Table 1, Fig. 1). After adjustment for age, sex, BMI and SBP, FGF19 levels still significantly decreased in order from NC, N-DM to DM (*p *< 0.001, Table 2). After adjustment for medications, the difference of serum FGF19 was significantly between the N-DM and DM groups (*p *< 0.05, Table 2). Compared with participants in the NC group, participants in the N-DM and DM groups were older and had higher BMI, SBP and FPG, TG (all *p* < 0.01) and LDL-c levels (*p* < 0.05) but lower HDL-c levels (*p* < 0.01). Additionally, individuals in the DM group exhibited higher FINS and HOMA-IR levels than subjects among N-DM group, but a lower level of ISSI-2 (*p* < 0.05; Table 1). Meanwhile, HbA1c did not differ between the N-DM and DM groups. Moreover, HbA1c, a reflection of 3-months glucose control was negatively correlated with serum FGF19 levels in DM group (r = − 0.247, *p* = 0.012; Fig. 2).

**Table 1:** Characteristics of the study participants

Variable	NC	N-DM	DM
n	64	85	122
Female n (%)	82.8	34.1^**^	43.4^**^
Age (years)	33.61 ± 10.64	51.38 ± 10.87^**^	53.15 ± 10.14^**^
BMI (kg/m^2^)	22.20 ± 2.59	25.41 ± 4.25^**^	25.60 ± 3.16^**^
W (cm)	N	92.62 ± 10.16	92.95 ± 9.82
SBP (mmHg)	110.3 (108.0–112.0)	137.2 (124.0–150.5)^**^	135.4 (127.0–145.0)^**^
DBP (mmHg)	90.1 (70.0–82.0)	83.8 (76.0–91.0)^*^	80.63 (74.0–89.0)^*^
FPG (mmol/L)	4.41 ± 0.30	5.97 ± 1.62^**^	5.81 ± 1.80^**^
FINS (mU/L)	N	112.0 (56.72–121.32)	160.19 (84.22–179.49)^##^
2hPG (mmol/L)	N	16.09 ± 3.98	16.81 ± 4.08
HbA1c (%)	N	9.48 ± 2.56	9.48 ± 2.44
HOMA-IR	N	28.92 (14.38–33.00)	41.55 (18.67–50.06)^#^
TC (mmol/L)	4.31 ± 0.79	4.55 ± 0.75	4.46 ± 1.04
TG (mmol/L)	1.21 ± 0.89	2.25 ± 2.05^*^	2.36 ± 2.23^**^
HDL-c (mmol/L)	1.34 ± 0.17	1.10 ± 0.28^**^	1.17 ± 0.32^**^
LDL-c (mmol/L)	2.52 ± 0.56	2.90 ± 0.68^*^	2.79 ± 0.93
FGF19 (pg/mL)	245.03 (126.23–317.43)	170.05 (89.01–244.70)^*^	142.25 (55.55–187.58)^**,#^
MI	N	9.39 ± 2.95	8.42 ± 3.58
AUC_ins/glu_	N	16.23 (6.92–19.43)	16.45 (7.09–20.93)
ISSI-2	N	146.44 (62.03–187.35)	122.94 (55.48–146.59)^#^

Categorical variables are frequency (percentage), normally distributed values in the table are mean ± SD and non-normally distributed values are median (25 and 75% interquartiles)

*BMI* body mass index, *W* waist, *SBP/DBP* systolic/diastolic blood pressure, *FPG* fasting plasma glucose, *FIN* fasting plasma insulin, *2hPG* 2-hour postprandial blood glucose, *HbA1c* glycosylated hemoglobin A1c, *HOMA-IR* homeostatic model assessment for insulin resistance, *TC* total cholesterol, *TG* triglyceride, *HDL-c* high density lipoprotein cholesterol, *LDL-c* low density lipoprotein cholesterol, *FGF19* fibroblast growth factor 19, *MI* Matsuda index, *AUCins/glu* the ratio of total area-under-the- insulin-curve to glucose-curve, *ISSI-2* Insulin Secretion-Sensitivity Index-2

p-values were determined using Student’s t-test, the Mann–Whitney U-test or the Chi-square test as appropriate

* p < 0.05, ** p < 0.01, the comparison of N-DM and DM with CN

^#^p < 0.05, ^##^p < 0.01, the comparison of N-DM and DM

**Table 2:** Univariate analysis of serum FGF19 among NC, N-DM and DM group

Group	Adjusted for age			Adjusted for s ex		
	Mean	95% CI	p	Mean	95% CI	p
NC	5.255	5.001–5.510		5.230	5.007–5.452	
N-DM	4.886	4.700–5.073	< 0.001	4.909	4.723–5.095	< 0.001
DM	4.589	4.428–4.751		4.587	4.434–4.740	

Group	Adjusted for BMI			Adjusted for SBP		
	Mean	95% CI	*p*	Mean	95% CI	*p*
NC	5.258	5.033–5.484		5.250	4.995–5.504	
N-DM	4.887	4.702–5.072	< 0.001	4.894	4.703–5.085	< 0.001
DM	4.590	4.434–4.747		4.587	4.429–4.745	

Group	Adjusted for medication		
	Mean	95% CI	*p*
N-DM	4.873	4.635–5.111	0.018
DM	4.491	4.299–4.682	

FGF19 did log transformation

p value < 0.05 was considered significant

### Serum FGF19 levels and beta cell function

**Fig. 3:**
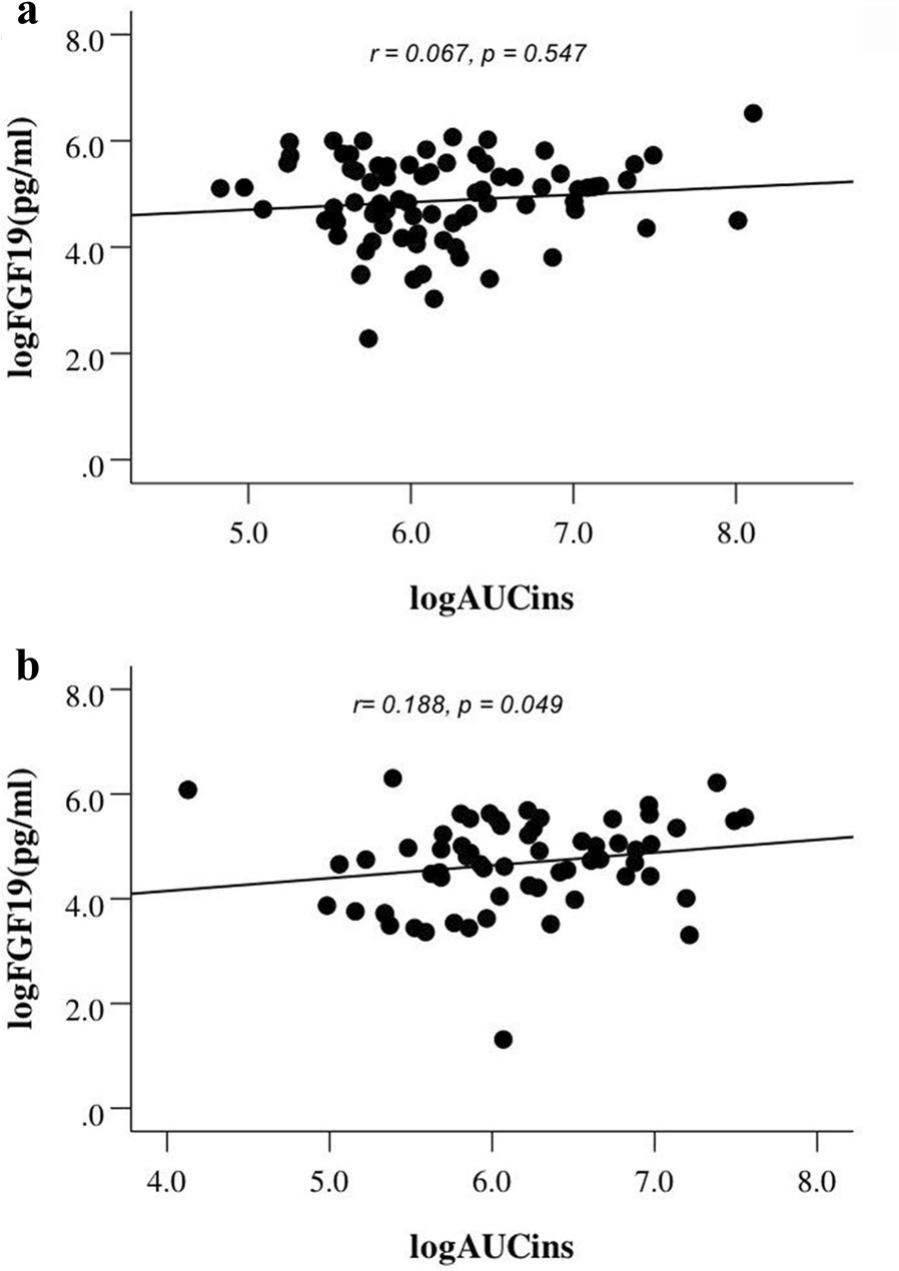
The relationship between AUCins and fasting FGF19 among N-DM (**a**) and DM (**b**)

**Fig. 4:**
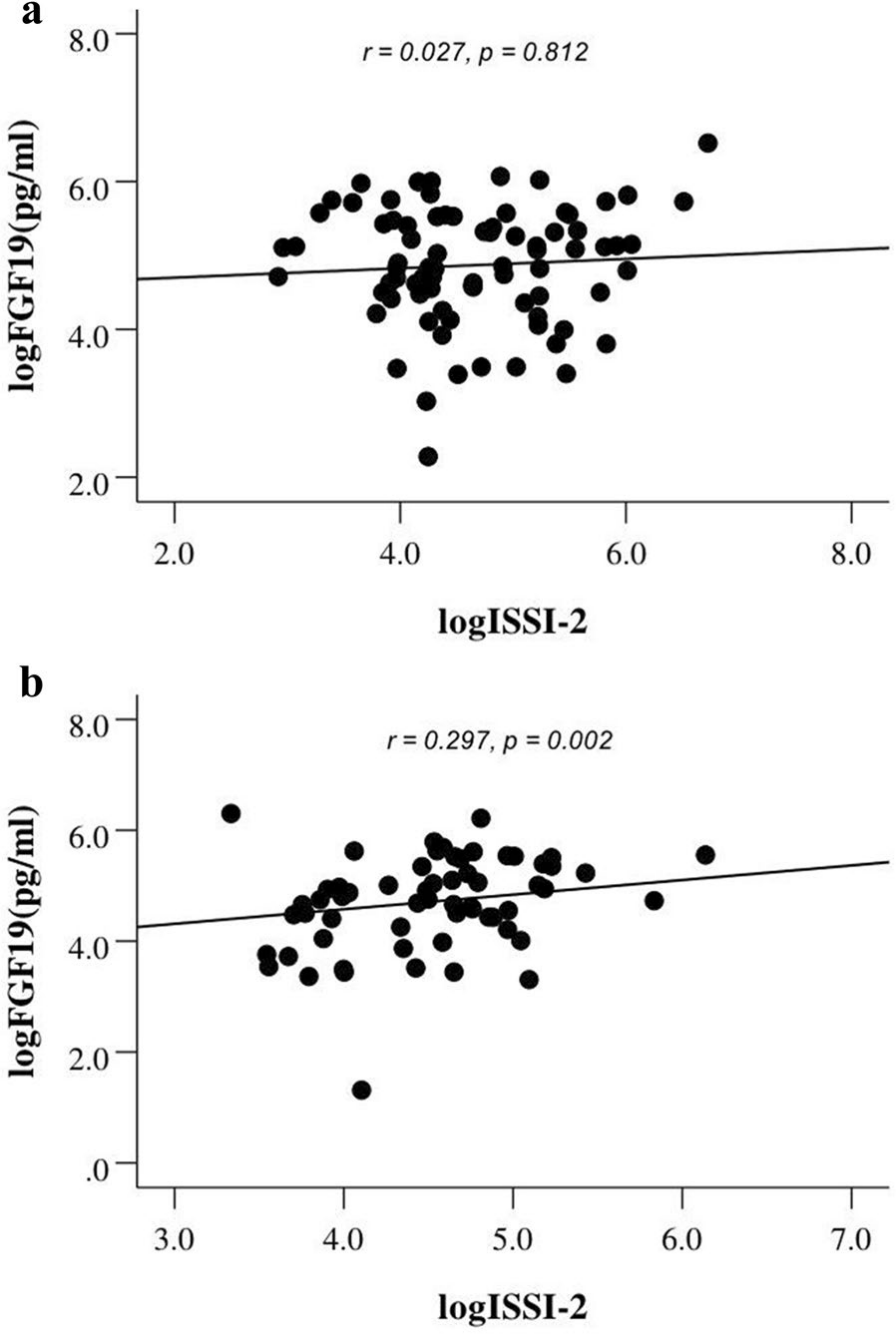
Correlation of ISSI-2 and fasting FGF19 among N-DM (**a**) and DM (**b**)

To detect the relationship between FGF19 and glucose, insulin, and beta cell function from the OGTT, Spearman analysis was performed. Serum FGF19 levels were positively correlated with insulin levels at 60 and 120 min after a glucose challenge in the DM group (*r *= 0.214, *p* = 0.025; *r* = 0.189, *p* = 0.048). Simultaneously, FGF19 level was positively correlated with the AUC_ins_ at two time points in the DM group (*r* = 0.188, *p* = 0.049; Fig. 3). Moreover, the MI, AUC_ins/glu_, and ISSI-2 among participants in DM group were analysed. Interestingly, only the AUC_ins/glu_ and ISSI-2 increased with increasing plasma FGF19 levels in the DM group (*r *=* 0.227, p *=* 0.018*; *r* = 0.297, *p* = 0.002; Fig. 4). However, no significant differences in AUC_ins/glu_ and ISSI-2 were observed in the N-DM group (*r* = 0.040, *p* = 0.720; *r* = 0.027, *p* = 0.812; Fig. 4).

### The determinants of FGF19 in DM group

To analyse whether serum FGF19 is independently correlated with insulin resistance, secretion, sensitivity and diabetes therapy, we performed multiple stepwise regression analysis. As is shown in Table 3, Model 1 considers gender, age, BMI, W, duration, hypertension, HbA1c, 2hPG, HOMA-IR, LDL-c, HDL-c, TG and TD. The results showed that ISSI-2 (*β *=* 0.321*, *t *=* 3.233*, *p *=* 0.002*, Table 3) was independently correlated with serum FGF19 levels. Based on Model 1, Model 2 further condensed the use of therapy (lifestyle intervention alone, insulin injection, insulin secretagogues and insulin sensitizers), and showed that W was independently and negatively connected with FGF19 levels. (*β *=− *0.220*, *t *=− *2.002*, *p *=* 0.049*, Table 3).

**Table 3:** Multivariate linear regression analyses on serum FGF19 levels

Model	Variable	β	t	p
Model 1	ISSI-2	0.321	3.233	0.002
Model 2	ISSI-2	0.281	2.557	0.013
	W	− 0.220	− 2.002	0.049

Original independent variables included

Model 1: gender, age, BMI, W, duration, hypertension, HbA1c, 2hPG, HOMA-IR, LDL-c, HDL-c, TG, TD

Model 2: Model 1+ antidiabetic treatments (lifestyle intervention alone, insulin injection, insulin secretagogues and insulin sensitizers)

## Discussion

In the current study, we compared FGF19 levels and investigated the relationship between FGF19 levels and islet beta cell function among a middle cohort of participants. The main findings of the present study are as follows. First, the serum concentrations of FGF19 were lower in N-DM group and even lower in DM group than in NC group regardless of the degree of insulin resistance. Second, serum FGF19 concentration was positively associated with INS60, INS120 level and the AUC_ins_ at two points. The AUC_ins/glu_ increased with increasing plasma FGF19 levels, but the correlation between ISSI-2 and FGF19 level was closer than the correlation between AUC_ins/glu_ and FGF19 level. However, these relationships were not significant in N-DM group. Third, after adjusting for sex, age, the use of therapy and other clinical factors via multiple logistic regression analysis, the ISSI-2 was still related to plasma FGF19 level. Additionally, as ISSI-2 is lower in DM group, FGF19 may be a crucial protector in dysfunction of islet beta cell.

Although the pancreas is an FGF19 target organ, data regarding the function of FGF19 in pancreatic islets remain scarce. Previous studies have detected that FGF19 plays an important role in keeping glucose homeostasis [8–11]. A study of the Chinese population demonstrated that the levels of fasting FGF19 were decreased in participants with decreased fasting glucose levels and naive diabetic patients and were inversely correlation with fasting glucose [5]. These data are agreement with our study. In addition, FGF19 could mediate its function via activating the FGFR4-β-Klotho complex [7]. Izaguirre et al. found that FGF19 combined with the FGFR4-β-Klotho complex to activate an insulin-independent endocrine pathway and mediate different metabolic effects [22]. Kir et al. reveal that FGF19 may play an insulin-like role in mouse liver and regulates hepatic glycogen via an insulin-independent mechanism [23]. Zhang et al. showed that FGF19 levels are positively associated with glucose effectiveness (GE) and negatively associated with hepatic glucose production (HGP) The increase in HGP in humans is partially due to the insulin-independent decrease in FGF19, which disagrees with our data [15]. ISSI-2 represents insulin sensitivity and insulin secretion during the OGTT. ISSI-2 and serum FGF19 levels were positively related in our research, which means ISSI-2 participates in insulin-dependent glucose regulation. Different durations of diabetes and treatments may cause these differences.

Previous studies have shown that when FGF19 was administered to diabetic mice in animal studies, plasma glucose levels were reduced, and glucose homeostasis was maintained [11]. These effects may be linked with glycogen synthase kinase 3 (GSK3), which is increased in hepatic glycogen storage [23] and inhibits the cyclic adenosine monophosphate regulatory element binding protein/peroxisome proliferator-activated receptor γ coactivator-1α-dependent (CREB/PGC1α) pathway [1]. Simultaneously, FGF19 contributed to weight loss and decreased glucose and insulin levels by β-Klotho in neurons [24].

Type 2 diabetes is associated with islet beta cell dysfunction. Fatty acid, hyperglycaemia, endoplasmic reticulum (ER) stress, or beta cell oxidative stress are factors that result in impaired islet beta cell function [25]. The present results suggest that serum FGF19 is independently correlated with insulin secretion and sensitivity and that increased serum FGF19 may improve the function of islet beta cells. However, in our study, the DM subjects were treated with insulin, insulin secretagogues and/or insulin sensitizers whose mechanisms of action are different. Wang et al. revealed that FGF19 serum decreased in DM rats and was continuously reduced after metformin treatment via AMPK-FXR crosstalk [26]. However, Sonne et al. showed no differences in FGF19 level in metformin-treated patients vs. patients treated with sulfonylureas and diet only [27]. We conducted univariate analysis and showed that serum FGF19 was significantly different between the N-DM and DM groups after adjustment for diabetic medications. Furthermore, multiple stepwise regression analysis was performed and showed that serum FGF19 was independently correlated with ISSI-2 in diabetic patients receiving treatment. However, further studies and larger sample sizes are needed to analyse the relationship between FGF19 and islet beta cell function in a sub-group of diabetic medications.

The mechanism of the relationship between FGF19 and islet beta cells remains unclear, but a possible explanation for this link is as follows. The circulating FGF19 level follows a diurnal rhythm depending on transintestinal BA flux after insulin secretion [28]. Isolated impaired fasting glucose (IFG), which depends on glucose metabolism, is due to impaired basal insulin secretion and increased HGP [29]. Therefore, individuals with isolated IFG have lower serum FGF19 levels than those without isolated IFG, suggesting that FGF19 plays a role in the secretion of impaired basal insulin [5]. The effect of the incretin GLP-1, a hormone that releases insulin by a G-protein coupled receptor, is dependent upon glucose [30]. The present study showed that GLP-1 secretion is inhibited by FXR through decreased glycolysis [16]. In addition, FXR^−^/^−^ mice are insulin resistant; their insulin levels were lower [31], and their islet size was smaller than those in control mice [32]. FGF19 clearly regulates BA homeostasis via the FXR-FGF19 axis [33]. Glucose homeostasis is also regulated by activation of FXR-TGR5 and GLP-1 secretion [16]. Our study reveals that serum FGF19 and ISSI-2 are lower in DM participants than in N-DM participants, which means that FGF19 may regulate islet β-cell secretion and insulin levels via the BA-FXR-FGF19 axis and GLP-1 secretion. The reason for this discrepancy in the serum levels of FGF19 in subjects with diabetes is unclear and requires exploration through further studies.

Several limitations in our study must be addressed. First, this was a cross-sectional design with medium sample sizes. This study could not explain the cause-effect connection between decreased serum FGF19 and islet beta cell secretion in diabetic patients. In other words, the secretion of islet beta cells may cause a decrease in the levels of serum FGF19. As this study was prospective, this weakness must be compensated for. Second, our research was conducted in Chinese participants, and the generalizability of our results should be evaluated. Third, our study consisted of the NC, N-DM, and DM groups; an increased number of different glucose tolerance states are warranted to assess the correlation between FGF19 levels and islet beta cell function. Fourth, ISSI-2 is closely related to FGF19 levels in the DM group. As the treatments of patients are different, the results may be influenced by therapies. However, serum FGF19 was still significantly different between the N-DM and DM groups after adjustment for medications and was independently associated with ISSI-2 after multiple stepwise regression analysis in DM group. Fifth, considering that previous studies showed that FGF19 was not significantly related with insulin sensitivity and secretion among NC subjects, only N-DM and DM groups underwent OGTT in our study; nonetheless, the correlation of FGF19 and islet beta cell function should also be observed in NC group. Therefore, further studies are needed to validate the results of the current study and address these limitations.

In summary, the serum FGF19 level is lower in DM subjects than in N-DM and NC subjects, but ISSI-2 is higher among the N-DM group. FGF19 may regulate endogenous islet beta cell function in diabetic patients with medical therapies.

## Conclusions

The serum FGF19 level has a close relationship with endogenous beta cell function among DM subjects, as assessed by the ISSI-2, but no significant association between serum FGF19 and integrated beta cell function was detected in the N-DM group. As ISSI-2 is higher in the N-DM group, FGF19 may be a main protector in beta cell dysfunction

## Data Availability

The current data are available to all interested researchers upon reasonable request. Requests for access to data should be made to principal investigators of the study, Meng-jie Tang (e-mail: 931736923@qq.com) and Xue-qin Wang (e-mail: wxqntdx@163.com).

## Abbreviations

BMI: body mass index
W: waist
SBP: systolic blood pressure
DBP: diastolic blood pressure
FPG: fasting plasma glucose
FIN: fasting plasma insulin
2hPG: 2-hour postprandial blood glucose
HbA1c: glycosylated haemoglobin A1c
HOMA-IR: homeostatic model assessment for insulin resistance
TC: total cholesterol
TG: triglyceride
HDL-c: high-density lipoprotein cholesterol
LDL-c: low-density lipoprotein cholesterol
FGF19: fibroblast growth factor 19
MI: Matsuda Index
AUC_ins/glu_: the ratio of the total area under the insulin curve to the total area under the glucose curve
ISSI-2: insulin secretion-sensitivity index-2
OGTT: oral glucose tolerance test
GE: glucose effectiveness
HGP: hepatic glucose production
GSK3: glycogen synthase kinase 3
CREB-PGC1α: cyclic adenosine monophosphate regulatory element binding protein/peroxisome proliferator-activated receptor γ coactivator-1α-dependent
GLP-1: glucagon-like peptide-1
ER: endoplasmic reticulum
